# Vision-Related Quality of Life following Combined Cataract and Minimally Invasive Glaucoma Surgery or Cataract Surgery Alone in Glaucoma Patients

**DOI:** 10.3390/jcm12093279

**Published:** 2023-05-04

**Authors:** Yuki Yuasa, Kazuyuki Hirooka, Naoki Okada, Hiromitsu Onoe, Yumiko Murakami, Hideaki Okumichi, Yoshiaki Kiuchi

**Affiliations:** 1 Department of Ophthalmology and Visual Science, Hiroshima University Graduate School of Biomedical Sciences, 1-2-3 Kasumi, Minami-ku, Hiroshima 734-8551, Japan; 2 Department of Ophthalmology, Hiroshima Prefectural Hospital, 1-5-54 Ujinakanda, Minami-ku, Hiroshima 734-8530, Japan

**Keywords:** glaucoma, vision-related quality of life, minimally invasive glaucoma surgery, cataract surgery, VFQ-25

## Abstract

This study examined glaucoma patients after undergoing combined cataract and minimally invasive glaucoma surgery (MIGS), microhook ab interno trabeculotomy and goniotomy with the Kahook Dual Blade (KDB), or cataract surgery alone, and it then evaluates their vision-related quality of life (VR-QOL) following the procedure. A total of 75 eyes of 75 consecutive glaucoma patients in this prospective cohort study underwent phacoemulsification (Phaco) or phaco and MIGS (Phaco-TLO) between October 2019 and March 2022. In all cases, the National Eye Institute Visual Function Questionnaire (VFQ-25) was used to evaluate the 20 eyes in the Phaco group and the 55 eyes in the Phaco-TLO group before and at 2 months after surgery. There was a significant increase in the visual acuity (logMAR) at 2 months post-operatively (Phaco group; 0.34 ± 0.10 to −0.07 ± 0.1, *p* < 0.0001, Phaco-TLO group; 0.37 ± 0.43 to 0.09 ± 0.32, *p* < 0.0001). The median (25–75th percentile) total VFQ scores in the Phaco group before and at 2 months after surgery were 71.1 (62.4–80.6) and 79.4 (69.0–84.0), respectively. (*p* = 0.006). The median (25–75th percentile) total VFQ scores in the Phaco-TLO group before and at 2 months after surgery were 69.8 (55.3–78.6) and 74.7 (65.1–83.3), respectively. (*p* = 0.005). Glaucoma patients who underwent not only cataract surgery alone but also combined cataract surgery and MIGS exhibited significant improvement in the VR-QOL.

## 1. Introduction

When intraocular pressure (IOP)-lowering medications do not effectively decrease the IOP during treatments of glaucoma, surgical treatment is necessary [1]. Glaucoma surgery lowers the IOP, thereby preventing the progression of visual field damage. Techniques for new surgical technologies, such as minimally invasive glaucoma surgeries (MIGS), have been developed, with these improvements helping to expand the types of procedures that can be performed during glaucoma surgical treatments [2].

In addition to the major glaucoma therapy and surgery target of maintaining the visual field perception and visual acuity, these also help to preserve the Quality of Life (QOL) of patients. To quantitatively measure vision-related QOL (VR-QOL), a 25-item scale, the National Eye Institute Visual Function Questionnaire (VFQ-25), has been developed [3]. There are 12 different domains that the VFQ-25 evaluates in order to measure the visual function, including general, color, driving, peripheral vision, the presence of ocular pain, visual function for near and distance activities, vision-specific social functioning, mental health, role difficulties, and dependency. In glaucoma patients, evaluations between the visual field disturbance due to glaucoma and the VR-QOL can be undertaken using the VFQ-25 [4,5,6]. Post-operative VR-QOL can potentially improve after cataract surgery [7]. The most commonly performed glaucoma surgery is trabeculectomy. In our previous study, we examined visual function both before and after trabeculectomy [8]. In order to minimize the IOP and the reliance on topical medications, it has been shown that the use of MIGS resulted in a rapid and safer visual recovery [2,9]. As the VR-QOL is influenced by cataract [10], the combined use of cataract surgery and MIGS should eventually lead to an improvement in the VR-QOL.

In the current study, we evaluated the VR-QOL in glaucoma patients after they underwent combined cataract and ab interno trabeculotomy (TLO), microhook ab interno trabeculotomy and goniotomy with the Kahook Dual Blade (KDB), or cataract surgery alone.

## 2. Materials and Methods

### 2.1. Patients

Between October 2019 and March 2022 at Hiroshima University Hospital, Japan, a total of 75 consecutive glaucoma patients underwent phacoemulsification (Phaco) or phaco-trabeculotomy (Phaco-TLO). The Institutional Review Board of the Hiroshima University approved this study protocol (E-1790). All subjects provided written informed consent in accordance with the principles outlined in the Declaration of Helsinki. Furthermore, prior to enrollment and participation in this research study, all patients provided standard consent for the surgery.

This prospective cohort study enrolled 20 patients in the Phaco group and 55 patients in the Phaco-TLO group. The National Eye Institute VFQ-25 scores were evaluated for changes between the results obtained before surgery and at 2 months after surgery. Visual field (VF) data were collected using program 24–2 of the Humphrey Field Analyzer (HFA) (Carl Zeiss Meditec, Dublin, CA, USA), with only reliable VF data utilized. During the analysis, HFA reliability criteria (<25% fixation losses, <15% false-positive errors) were applied. Exclusion criteria included patients who were less than 20 years of age, had the presence of dementia, or a history of intraocular surgery. Patients were also excluded if there were conditions ranging from secondary glaucoma to uveitis or neovascular glaucoma. We also excluded the eyes of patients with a history of glaucoma surgery.

### 2.2. Outcome

The VFQ-25 was completed by patients both before and at 2 months after surgery. As previously mentioned, there were 12 subscales that the items were assigned to and which included general health and vision, the presence of ocular pain, the visual function for near and distance activities, the vision-specific social functioning, mental health, role difficulties, dependency, driving, and color and peripheral vision. Subscales scores ranged from 0 to 100 points, with the highest possible function or minimal subjective impairment defined as being 100. The VFQ-25 utilized in this study was the Japanese version of the questionnaire, but which included a few modifications that were completed to take into account the Japanese culture and lifestyle.

Changes in the best corrected visual acuity (BCVA, logMAR), changes in the IOP, and changes in IOP-lowering medications score were used as the secondary endpoints. In order to evaluate the scores for the IOP-lowering medications, a single drug was defined as a score of 1 point while a combination of drugs and oral drugs was defined as a score of 2 points.

Transient ocular hypertension was defined as a postoperative IOP elevation > 30 mmHg. Hyphema was defined as hemorrhages in the anterior chamber with niveau formation.

### 2.3. Surgical Procedure

There were multiple surgeons who performed the surgeries, with all of the surgical methods generally the same. All cataract surgeries were performed using a temporal 2.8 mm corneal incision. The cataract instrument used was the Signature Pro (Johnson & Johnson, New Brunswick, NJ, USA), with the PCB00V (Johnson & Johnson) or XY1 (HOYA, Tokyo, Japan) intraocular lens then implanted in the capsular bag. Sufficient sodium hyaluronate (Healon^®^, Johnson & Johnson Vision, Irvine, CA, USA) was added into the anterior chamber in order to increase the visuality of the Schlemm’s canal after the cataract surgery. Subsequently, after tilting the patient’s head and microscope, the Hill lens (Ocular Inst., Bellevue, WA, USA) was placed on the cornea. After then identifying the location of Schlemm’s canal, the microhook (Inami & Co., Ltd., Tokyo, Japan) or the KDB (New World Medical, Rancho, Cucamonga, CA, USA) was inserted through the main incision. A nasal and inferior site incision was subsequently made between 120° and 180°. The degree of the incision and the type of antibacterial and anti-inflammatory eye drops were prescribed in line with the personal preferences of each of the surgeons. At the time of the initial surgery, all of the IOP-lowering medications were stopped, with the resumption of the medications at post-operative follow-up visits determined in accordance to the discretion of the surgeon.

### 2.4. Statistical Analyses

Using a significance level of 5% (α) and a power of 80% (1-β), the pre-study power calculation determined that a sample size of 50 patients would be required in order to detect a median VFQ-25 composite score difference of 4 points in the Phaco-TLO group. Furthermore, these calculations indicated that in order to detect a median VFQ-25 composite score difference of 8 points in the Phaco group, a sample size of 15 patients would be required. Subsequently, we then calculated the median scores for each of the VFQ-25 subscales and the composite score. A Wilcoxon signed-rank test was used to compare the pre- and post-operative results. Determination of the significance of the comparison between the Phaco and Phaco-TLO groups was performed using a Mann–Whitney U test and chi-square test. All statistical analyses used JMP software, version 16 (SAS Inc, Cary, NC, USA), with *p* < 0.05 considered to be significant.

## 3. Results

Baseline patient data are presented in Table 1. In 20 eyes (Phaco group), cataract surgery was performed, while in 55 eyes (Phaco-TLO group), combined cataract surgery with TLO was performed. The baseline IOPs in the Phaco and Phaco-TLO groups were 12.5 ± 1.1 mmHg and 16.1 ± 0.7 mmHg, respectively (*p* = 0.002), while the IOP-lowering medications scores were 1.7 ± 0.3 and 2.7 ± 0.2, respectively (*p* = 0.002). For the visual field index, the values for the Phaco and Phaco-TLO groups were 83.5 ± 12.9% and 67.7 ± 27.1%, respectively (*p* = 0.048). There were no significant differences observed between the groups for age, gender, type of glaucoma, visual acuity, and mean deviation.

The comparison of the changes in the visual acuity before and at 2 months after the surgery for the IOP and IOP-lowering medications score are presented in Table 2. There was significant improvement in the BCVA observed in both groups. Although in the Phaco-TLO group there was a significant difference noted for the IOP and IOP-lowering medications score, in the Phaco group, no significant differences were observed for the IOP. Subsequently, we then performed a comparison between the Phaco and Phaco-TLO groups in order to evaluate the real impact of these surgeries. No significant differences were noted for the BCVA, IOP, or IOP-lowering medications score between the two groups (*p* = 0.16, 0.26, 0.29, Mann–Whitney U test).

Table 3 presents a comparison of the VFQ scores in the Phaco group before and at 2 months after surgery. As compared to the preoperative scores for the general vision, near and distant visual acuity, social functioning, mental health, and composite score, the postoperative scores were significantly higher. There was an increase of the near visual acuity score in 16 patients (80%), while the distant visual acuity score increased in 13 patients (65%). The results for the pre- and postoperative VFQ-25 composite scores and the 12 subscales that were found in the patients who underwent combined cataract and TLO ab interno are presented in Table 4. For the VFQ-25 questionnaire scales that examined the general vision, near and distant visual acuity, mental health, and composite score, significantly higher results were found for the post-operative versus the pre-operative values. The near visual acuity score increased in 31 patients (56%), while the distant visual acuity score increased in 32 patients (58%). There were no significant differences noted between the Phaco and Phaco-TLO groups for the pre- (*p* = 0.054 to 0.79) and postoperative (*p* = 0.056 to 0.80) VFQ-25 composite scores and each of the 12 subscales.

In the Phaco group, there were two eyes, while there were six eyes in the Phaco-TLO group that exhibited post-operative transient ocular hypertension. In four eyes of the Phaco-TLO group, there was hyphema with niveau formation.

## 4. Discussion

This study evaluated the VR-QOL of glaucoma patients before and after undergoing combined cataract and MIGS. When comparisons between the Phaco and Phaco-TLO groups were conducted for values before and 2 months after surgery, there was significant improvement in the visual acuity noted in both groups. Furthermore, there was significant improvement in the Phaco and Phaco-TLO groups at 2 months after surgery for the composite score of VFQ-25.

Several studies have examined the VR-QOL following glaucoma surgery. Hirooka et al. [8] evaluated the VR-QOL in glaucoma patients and reported that although there was no improvement noted after glaucoma filtration surgery, patients who underwent combined cataract and glaucoma filtration surgery did show a significant improvement in the VR-QOL. Moreover, at 6 months after surgery, Pahlitzsch et al. [11] reported that the VFQ-25 findings indicated that there was no statistically significant difference between trabeculectomy and MIGS. Another recent report by Habash et al. [12] that investigated glaucoma patients who underwent MIGS, which included KDB goniotomy, iStent, iStent inject, and gonioscopy-assisted transluminal trabeculotomy, found that there was improvement in the VR-QOL when evaluated from different perspectives. Furthermore, they also found that there was a reduction in the number of IOP-lowering medications that were needed, thereby helping to reduce the associated side effects. A further study that evaluated subjects who underwent cataract surgery alone as compared to subjects who underwent cataract surgery plus iStent inject implantation, found there was a much greater improvement for the VR-QOL in the latter group [13]. However, it should be noted that with the exception for Hirooka et al., the VR-QOL was only evaluated after the patients underwent the surgery [11,12,13]. Thus, the degree of the improvement for VR-QOL after surgery has yet to be definitively established.

Additional studies that have investigated patients undergoing cataract surgery in which there were no ocular diseases with the exception of cataracts have also reported finding a significantly improved VFQ-25 score [14,15]. The predictive factor for post-operative VR-QOL improvement has been reported to be a low pre-operative VR-QOL and the BCVA [14]. In a cross-sectional study by Suzukamo et al. [16], the authors reported that the visual acuity was strongly correlated with the VFQ-25 composite score. Similarly, our current study also found that there was a significant improvement in the BCVA in both the Phaco and the Phaco-TLO group. In our current study, we also found that even in moderate to mild glaucoma patients, after the cataract surgery there was an improvement in the composite score.

Suzukamo et al. [16] investigated the VFQ-25 subscale, which is known to be less dependent on central vision, including general health, peripheral vision, ocular pain, and color vision subscales, and reported that visual acuity was only weakly correlated with this scale. Makabe et al. [14] additionally evaluated other items such as social function, ocular pain, and general health subscales, and reported that changes in some of these scales were not correlated with visual acuity improvements that were noted after cataract surgery. Our present study results for both Phaco and Phaco-TLO groups were similar to these previous findings.

The local and systemic side effects associated with IOP-lowering medication are well known and through the reduction of the IOP, they help to improve the patient’s VR-QOL [17]. As conjunctival hyperemia and eye pain can be side effects of eye drops, it is logical to assume that a decrease in the IOP-lowering medications score would likely lead to a reduction followed by a subsequent improvement in the ocular surface discomfort. In fact, there was a significant decrease in the IOP-lowering medications score in our current study for the Phaco-TLO group. As a result, this may lead to a significant increase in the composite score of VFQ-25.

However, it should be noted that it has been reported by some investigators that in conjunction with a worsening of the glaucomatous visual field defects, there was also a decrease in the VR-QOL [18,19,20]. Hirooka et al. [20] additionally found that if the visual field index (VFI) was <50% in the worse eye of a glaucoma patient, there was a significant reduction in the VR-QOL. In our Phaco-TLO group, we found that the VFI was <50% in 11 (20%) eyes. In contrast, in the Phaco group, we found the VFI to be ≥50% in all eyes. Therefore, due to glaucomatous visual field defects that were found in some of the patients in our Phaco-TLO group, this may be the reason for the decrease in the VR-QOL. In fact, in our Phaco-TLO group, the VFQ-25 composite score tended to be lower as compared to that found in the Phaco group. Thus, the significant differences in the VFI found between the two groups may affect the VR-QOL.

In our current study, there were some limitations. First, the VR-QOL evaluations were undertaken at 2 months after surgery. In one of our recent studies that investigated corneal higher-order aberrations (HOAs) and coma-like or spherical-like aberrations, we found that for up to 3 months after the Phaco-TLO procedure, these aberrations remained significantly increased from the baseline [21]. Thus, the results of the VFQ-25 could have been affected by the corneal HOAs and coma-like or spherical-like aberrations. Therefore, long-term follow-up will need to be undertaken in future studies in order to confirm these initial findings. However, when patients are evaluated using the VR-QOL, the possibility of an effect caused by glaucoma progression cannot be ignored, especially when the evaluations are undertaken after an extended period of time has passed since the surgery. The second limitation is that the degree of the pre-operative cataract was not evaluated, such as using the Lens Opacities Classification System (LOCS) III. A previous study that evaluated participants with posterior subcapsular opacity reported finding an association between lens opacity and VR-QOL [22]. In contrast, it may be that there is no relationship between the LOCS III grading and the VFQ-25 composite score, but, rather, there may be an association with some of the VFQ-25 subscales [14]. Furthermore, other factors besides the visual acuity could influence the VR-QPL, including contrast sensitivity and aberration. The third limitation is that there was a significant difference in the baseline score for the IOP-lowering medications and the glaucoma stage between the Phaco and the Phaco-TLO group. These differences could have potentially affected the results of the study. However, the main aim of our current study was to compare the changes in each group before and after the surgery, and not to perform comparisons of the overall changes between the Phaco and the Phaco-TLO groups. The forth limitation is that evaluation of VR-QOL took place at two months after the surgery.

In conclusion, after patients underwent combined cataract and ab interno TLO, microhook ab interno trabeculotomy, and goniotomy with the KDB, significant improvements in the VR-QOL were observed.

## Figures and Tables

**Table 1:** Baseline characteristics of the Phaco and Phaco-TLO groups.

	Phaco (*n* = 20)	Phaco-TLO (*n* = 55)	*p* Value
Age (years)	73.3 ± 7.4	71.5 ± 9.7	0.51
Gender (Male/Female)	10/10	21/34	0.36
Type of glaucoma			0.49
Primary open-angle glaucoma	14	29	
Primary angle-closure glaucoma	5	17	
Exfoliation glaucoma	1	8	
Steroid glaucoma	0	1	
Visual acuity (logMAR)	0.34 ± 0.42	0.37 ± 0.43	0.89
IOP (mmHg)	12.5 ± 2.8	16.1 ± 5.4	0.002
IOP-lowering medications score	1.7 ± 1.4	2.7 ± 1.2	0.002
Mean deviation (dB)			
Operated eye	−8.7 ± 4.3	−11.9 ± 7.0	0.11
Fellow eye	−8.5 ± 7.1	−10.2 ± 9.3	0.54
Visual field index (%)			
Operated eye	83.5 ± 12.9	67.7 ± 27.1	0.048
Fellow eye	77.9 ± 23.2	70.7 ± 26.7	0.62

IOP; intraocular pressure.

**Table 2:** Change in visual acuity, IOP, and IOP-lowering medications before and after surgery.

		Phaco			Phaco-TLO	
	Before	After	*p* Value	Before	After	*p* Value
BCVA	0.34 ± 0.42	−0.02 ± 0.07	<0.0001	0.37 ± 0.43	0.09 ± 0.32	<0.0001
IOP (mmHg)	12.5 ± 2.8	11.6 ± 2.9	0.41	16.1 ± 5.4	12.6 ± 2.8	<0.0001
IOP-lowering medications	1.7 ± 1.4	0.9 ± 1.3	0.002	2.7 ± 1.2	1.0 ± 0.9	<0.0001

BCVA; best corrected visual acuity, IOP; intraocular pressure.

**Table 3:** Comparison of before and at 2 months after surgery VFQ scores in the cataract surgery group (*n* = 20).

VFQ-25 Scale	Preoperative (IQR)	Postoperative (IQR)	*p* Value
General health	60.0 (45.6–69.4)	60.0 (51.3–71.9)	0.15
General vision	60.0 (50.0–65.0)	75.0 (61.3–80.0)	0.009
Ocular pain	87.5 (70.0–100)	87.5 (70.0–100)	0.43
Near visual acuity	62.5 (58.3–75.0)	75.0 (63.5–83.3)	0.0007
Distant visual acuity	70.8 (58.3–78.1)	77.1 (64.6–83.3)	0.046
Social functioning	79.2 (75.0–83.3)	83.3 (75.0–91.7)	0.04
Mental health	65.0 (51.3–80.0)	80.0 (71.3–93.8)	0.01
Role difficulties	81.3 (68.8–98.4)	87.5 (76.6–100)	0.37
Dependency	93.8 (70.3–100)	100 (79.7–100)	0.51
Driving	66.7 (50.0–83.3)	75.0 (66.7–83.3)	0.25
Color vision	100 (75.0–100)	100 (100–100)	0.06
Peripheral vision	0 (0–75.0)	12.5 (0–75.0)	>0.99
Composite score	71.1 (62.4–80.6)	79.4 (69.0–84.0)	0.006

IQR; interquartile range, VFQ; Visual Function Questionnaire. *p* values were calculated using Wilcoxon signed-rank test.

**Table 4:** Comparison of before and at 2 months after surgery VFQ scores in the Phaco-TLO group (*n* = 55).

VFQ-25 Scale	Preoperative (IQR)	Postoperative (IQR)	*p* Value
General health	55.0 (42.5–65.0)	55.0 (42.5–65.0)	0.89
General vision	55.0 (45.0–65.0)	70.0 (55.0–80.0)	<0.0001
Ocular pain	75.0 (62.5–87.5)	75.0 (62.5–87.5)	0.14
Near visual acuity	70.8 (50.0–79.2)	75.0 (62.5–83.3)	0.002
Distant visual acuity	66.7 (54.2–75.0)	75.0 (62.5–79.2)	0.02
Social functioning	83.3 (75.0–91.7)	83.3 (66.7–91.7)	0.63
Mental health	65.0 (50.0–85.0)	75.0 (55.0–90.0)	0.002
Role difficulties	75.0 (62.5–100)	81.3 (68.8–100)	0.08
Dependency	87.5 (68.8–100)	87.5 (75.0–100)	0.41
Driving	70.8 (47.9–75)	75.0 (58.3–83.3)	0.15
Color vision	100 (75.0–100)	100 (75.0–100)	0.96
Peripheral vision	37.5 (0–75.0)	50.0 (0–75.0)	0.21
Composite score	69.8 (55.3–78.6)	74.7 (65.1–83.3)	0.005

IQR; interquartile range, *p* values were calculated using Wilcoxon signed-rank test.

## Data Availability

The data analyzed in this study are available from the corresponding author on reasonable request.

## References

[1] Aoyama A., Ishida K., Sawada A., Yamamoto T. (2010). Target intraocular pressure for stability of visual field loss progression in normal-tension glaucoma. Jpn. J. Ophthalmol..

[2] Francis B.A., Singh K., Lin S.C., Hodapp E., Jampel H.D., Samples J.R., Smith S.D. (2011). Novel glaucoma procedures: A report by the American Academy of Ophthalmology. Ophthalmology.

[3] Mangione C.M., Lee P.P., Gutierrez P.R., Spritzer K., Berry S., Hays R.D. (2001). Development of the 25-item National Eye Institute visual function questionnaire. Arch. Ophthalmol..

[4] McKean-Cowdin R., Wang Y., Wu J., Azen S.P., Varma R., Los Angeles Latino Eye Study Group (2008). Impact of Visual Field Loss on Health-Related Quality of Life in Glaucoma: The Los Angeles Latino Eye Study. Ophthalmology.

[5] Spaeth G., Walt J., Keener J. (2006). Evaluation of quality of life for patients with glaucoma. Am. J. Ophthalmol..

[6] Lisboa R., Chun Y.S., Zangwill L.M., Weinreb R.N., Rosen P.N., Liebmann J.M., Girkin C.A., Medeiros F.A. (2013). Association between rates of binocular visual field loss and vision-related quality of life in patients with glaucoma. JAMA Ophthalmol..

[7] Hiratsuka Y., Yamada M., Akune Y., Murakami A., Okada A.A., Yamashita H., Ohashi Y., Yamagishi N., Tamura H., Fukuhara S. (2014). Assessment of vision-related quality of life among patients with cataracts and the outcomes of cataract surgery using a newly developed visual function questionnaire: The VFQ-J11. Jpn. J. Ophthalmol..

[8] Hirooka K., Nitta E., Ukegawa K., Tsujikawa A. (2017). Vision-related quality of life following glaucoma filtration surgery. BMC Ophthalmol..

[9] Tanito M., Ikeda Y., Fujihara E. (2017). Effectiveness and safety of combined cataract surgery and microhook ab interno trabeculectomy in Japanese eyes with glaucoma: Report of an initial case series. Jpn. J. Ophthalmol..

[10] Skalicky S.E., Martin K.R., Fenwick E., Crowston J.G., Goldberg I., McCluskey P. (2015). Cataract and quality of life in patients with glaucoma. Clin. Exp. Ophthalmol..

[11] Pahlitzsch M., Klamann M.K., Pahlitzsch M.L., Gonnermann J., Torun N., Bertelmann E. (2017). Is there a change in the quality of life comparing the micro-invasive glaucoma surgery (MIGS) and the filtration technique trabeculectomy in glaucoma patients?. Graefes Arch. Clin. Exp. Ophthalmol..

[12] Al Habash A., Nagshbandi A.A. (2020). Quality of life after combined cataract and minimally invasive glaucoma surgery in glaucoma patients. Clin. Ophthalmol..

[13] Samuelson T.W., Singh I.P., Williamson B.K., Falvey H., Lee W.C., Odom D., McSorley D., Katz L.J. (2021). Quality of life in primary open-angle glaucoma and cataract: An analysis of VFQ-25 and OSDI from the iStent inject^®^ pivotal trial. Am. J. Ophthalmol..

[14] Manabe K., Oshika T., Inamura M., Hayashi K., Sugita G., Kozawa T., Fujishima K. (2020). Influence of cataract surgery for the first or second eye on vision-related quality of life (VR-QOL) and the predictive factors of VR-QOL improvement. Jpn. J. Ophthalmol..

[15] Li X., Lin J., Chen Z., Jin G., Zheng D. (2021). The impact of cataract surgery on vision-related quality of life and psychological distress in monocular patients. J. Ophthalmol..

[16] Suzukamo Y., Oshika T., Yuzawa M., Tokuda Y., Tomidokoro A., Oki K., Mangione C.M., Green J., Fukuhara S. (2005). Psychometric properties of the 25-item national eye institute visual function questionnaire (NEI VFQ-25), Japanese version. Health Qual. Life Outcomes.

[17] Meier-Gibbons F., Töteberg-Harms M. (2020). Influence of cost of care and adherence in glaucoma management: An update. J. Ophthalmol..

[18] Parrish P.K., Gedde S.J., Scott L.U., Feuer W.J., Schiffman J.C., Mangione C.M., Montenegro-Piniella A. (1997). Visual function and quality of life among patients with glaucoma. Arch. Ophthalmol..

[19] Sherwood M.B., Garcia-Siekavizza A., Meltzer M.l., Hebert A., Burns A.F., McGorray S. (1998). Glaucoma’s impact on quality of life and its relation to clinical indicators. A pilot study. Ophthalmology.

[20] Hirooka K., Sato S., Nitta E., Tsujikawa A. (2016). The relationship between vision-related quality of life and visual function in glaucoma patients. J. Glaucoma.

[21] Onoe H., Hirooka K., Okumichi H., Murakami Y., Kiuchi Y. (2021). Corneal higher-order aberrations after microhook ab interno trabeculotomy and coniotomy with the Kahook Dual Blade: Preliminary early 3-month results. J. Clin. Med..

[22] Wu S.Y., Hennis A., Nemesure B., Leske M.C., Barbados Eye Studies Group (2008). Impact of glaucoma, lens opacities, and cataract surgery on visual functioning and related quality of life: The Barbados Eye Study. Investig. Ophthalmol. Vis. Sci..

